# Long COVID and chronic fatigue syndrome/myalgic encephalitis share similar pathophysiologic mechanisms of exercise limitation

**DOI:** 10.14814/phy2.70535

**Published:** 2025-09-02

**Authors:** Swathi Jothi, Michael Insel, Guido Claessen, Saad Kubba, Erin J. Howden, Sergio Ruiz‐Carmona, Todd Levine, Franz P. Rischard

**Affiliations:** ^1^ Division of Pulmonary, Critical Care, Sleep, and Allergy Medicine University of Arizona Tucson Arizona USA; ^2^ Department of Cardiovascular Sciences KU Leuven Leuven Belgium; ^3^ Sarver Heart Center, University of Arizona Tucson Arizona USA; ^4^ Baker Heart and Diabetes Institute Melbourne Australia; ^5^ Honor Health Neurology Scottsdale Arizona USA

**Keywords:** chronic fatigue syndrome, exercise, long‐COVID, myalgic encephalitis, post‐acute sequelae of SARS Co‐V2

## Abstract

Post‐acute sequelae of SARS‐CoV‐2 (PASC or “long COVID”) and chronic fatigue syndrome/myalgic encephalitis (CFS/ME) share symptoms such as exertional dyspnea. We used exercise oxygen pathway analysis, comprising six parameters of oxygen transport and utilization, to identify limiting mechanisms in both conditions. Invasive cardiopulmonary exercise testing was performed on 15 PASC patients, 11 CFS/ME patients, and 11 controls. We evaluated the contributions of alveolar ventilation (V̇a), lung diffusion capacity (D_L_ ), cardiac output (Q̇), skeletal muscle diffusion capacity (D_M_ ), hemoglobin (Hb), and mitochondrial oxidative phosphorylation (V_max_) to peak oxygen consumption (V̇O_2peak_). To simulate targeted interventions, each variable was sequentially normalized to assess its impact on V̇O_2peak_. V̇O_2peak_ was significantly reduced in both PASC and CFS/ME compared to controls. Skeletal muscle O_2_ diffusion (D_M_ ) was the most impaired parameter in both patient groups (*p* = 0.01). Correcting D_M_ alone improved V̇O_2_ by 66% in PASC (*p* = 0.008) and 34.7% in CFS/ME (*p* = 0.06), suggesting a dominant role for peripheral O_2_ extraction in exercise limitation. Impaired skeletal muscle oxygen diffusion (D_M_ ) is a shared mechanism of exercise intolerance in PASC and CFS/ME and may represent a therapeutic target. However, our findings are limited by small sample size.

## 1 INTRODUCTION

Persistent symptoms after acute COVID‐19 are common and significantly impact patient quality of life (Raman et al., 2021). Estimates indicate that over 7% of the U.S. adult population has the condition (17.8 million) (Fang et al., 2024). Patients with prolonged (>3 months (Groff et al., 2021)) symptoms after acute SARS CoV‐2 infection, termed Post‐Acute sequelae of SARS CoV‐2 (PASC) or “Long COVID” suffer from persistent and unexplained dyspnea (24%–87%), post‐exertional fatigue (58%–98%), and a multitude of other symptoms (Davis et al., 2020; Huang, Huang, et al., 2021a; Huang, Pinto, et al., 2021b; Lopez‐Leon et al., 2021; Thaweethai et al., 2023). PASC is accompanied by abnormalities in standard tests such as lung imaging, pulmonary function testing, and gas exchange (arterial blood gases) (Dennis et al., 2020). However, there is poor agreement between these standard resting tests and exertional fatigue and dyspnea (Aparisi et al., 2021; Froidure et al., 2021; Huang, Huang, et al., 2021a; Rinaldo et al., 2021). The clinical importance of persistent symptoms is underscored by a link to patient reported outcomes (PROs) (Aparisi et al., 2021; Davis et al., 2020; Raman et al., 2021) and a reduction in peak oxygen consumption (V̇O_2peak_) (Raman et al., 2021; Ybarra‐Falcón et al., 2021).

Studies employing cardiopulmonary exercise testing (CPET), used to measure V̇O_2peak_, have proposed heterogeneous underlying mechanisms including deconditioning (Edward et al., 2023; Rinaldo et al., 2021), hyperventilation (Motiejunaite et al., 2021), “dysfunctional breathing” (Mancini et al., 2021), and abnormal peripheral neuromuscular response (Singh et al., 2021). The majority of these studies have used noninvasive CPET (nCPET) which only provides hints of mechanistic causes of observed abnormalities. Unlike nCPET, invasive CPET (iCPET), which is nCPET with a pulmonary artery catheter and arterial line, provides comprehensive physiologic readouts and more granular information such as cardiac output and peripheral oxygen extraction to define sub‐phenotypes of PASC.

Many patients with PASC share symptomatology with chronic fatigue syndrome/myalgic encephalitis (CFS/ME) leading experts to consider potential shared pathophysiology (Herrera et al., 2021). Both syndromes in some patients appear to be associated with dysautonomia, possibly related to small fiber neuropathy (SFN), resulting in a low cardiac preload state exacerbating dyspnea (Joseph et al., 2021; Risbano et al., 2023). Both conditions are also associated with impaired oxygen extraction during exercise testing (Joseph et al., 2021; Singh et al., 2021; Vermeulen & Vermeulen van Eck, 2014). However, one factor underlying the conclusion that oxygen extraction is a primary pathophysiological target is the erroneous assumption that oxygen extraction is independent of cardiac output. In the human body, exercise peripheral O_2_ extraction is dependent on oxygen delivery as well as skeletal muscle capillary transit time, and changes in these metrics may be a result of their dependency rather than any direct physiological insult (Houstis et al., 2018; Wagner, 2011).

To more accurately assess the underlying mechanisms of reduced V̇O_2peak_, cardiac output and O_2_ extraction should be measured separately rather than with traditional methods of exercise analysis using the Fick method. Oxygen pathway analysis (Houstis et al., 2018; Wagner, 2011) obtained from iCPET uses separate equations for each component related to V̇O_2_ from mouth to muscle. There has been success applying this method to heterogeneous phenotypes such as heart failure with preserved ejection fraction. This approach adds a level of granularity to identify potential therapeutic targets. By personalizing targets relative to established norms, we can quantify the magnitude (and potential benefit) of a treatment target.

The aims of this analysis are (1) to confirm a physiologic insult in PASC and CFS/ME patients relative to controls, (2) describe the overall similarities and differences in traditional exercise variables between CFS/ME and PASC, and (3) to apply formal oxygen pathway analysis to both conditions to illustrate potential pathophysiological similarities and differences as well as potential treatment targets.

## 2 METHODS

### 2.1 Subjects

The study participants comprised 15 PASC patients, 11 CFS/ME patients, and 11 controls. Acute/post‐acute (>3 months) SARS‐CoV‐2 infected patients with dyspnea (mMRC ≥ 1) and/or severe post‐exertional fatigue made up the PASC arm of the study. All PASC participants met World Health Organization probable or confirmed criteria for COVID‐19 infection (World Health Organization, 2022). Subjects in the CFS/ME cohort met all major and at least one minor Institute of Medicine criteria (Committee on the Diagnostic Criteria for Myalgic Encephalomyelitis/Chronic Fatigue Syndrome et al., 2015). The CFS/ME patients included in this analysis data was obtained prior to March 2020 (the COVID epidemic) and thus had not been infected with SARS‐CoV‐2 at the time of assessment.

The control arm comprised of two groups; one evaluated in our pulmonary hypertension clinic for dyspnea or echocardiographic evidence of PH and a subsequent normal iCPET and individuals in good health recruited from a study in Belgium. All our control group participants were considered “healthy” by virtue of a peak exercise capacity and cardiac output ≥80% predicted, respiratory exchange ratio (RER) >1.05, and had objective evidence of normal cardiovascular function, defined as normal ECG and transthoracic echocardiogram (left ventricular EF >50%), and normal pulmonary artery pressures at rest (<25 mm Hg) and during exercise (slope of mean pulmonary artery pressure to cardiac output <3 mm Hg/[L·min]). Ethical approval for this study was obtained from the University of Arizona Institutional Review Board (IRB# 1100000621) and written informed consent was obtained from all participants included in the study.

### 2.2 Standard and invasive cardiopulmonary exercise testing

After study inclusion, subjects underwent standard testing for their dyspnea as directed by evaluating pulmonary and/or cardiologists. All subjects had pulmonary function testing, chest x‐ray, and echocardiography. CT chest scans were performed in all CFS/ME and 12 PASC subjects. Cardiac MRI was done in 3 PASC subjects. Ventilation/perfusion SPECT scans were obtained in 4 PASC subjects.

iCPET testing was done on all subjects with a pulmonary artery catheter that was advanced via the antecubital vein after ultrasonic access for measurements of pulmonary artery pressure (systolic, mean, and diastolic PAP), RV pressure, right atrial pressure (RAP), wedged PAP (PCWP), and cardiac output (direct Fick). Radial arterial lines were placed also under ultrasonic guidance. Our comprehensive resting and exercise catheterization protocol has been previously published (Tang et al., 2020). Briefly, after obtaining supine resting measurements, the patient was placed in a full upright position with an electronic fluoroscopy chair. Fluoroscopy was used to re‐zero at left atrial level (Kovacs et al., 2014). A cycle ergometer was positioned below the patient. The patient then proceeded with exercise at a predetermined workload based on their level of dyspnea (Tang et al., 2020) for steady‐state 2‐min stages until RER ~ 1.1. PAP, PCWP, RAP, arterial and venous O_2_ content (CaO_2_ and CvO_2_) were obtained during the last 30 s of each stage. Metabolic cart analysis (Vyaire Medical™, Mettawa, IL) was used for simultaneous collection of gas exchange and lung volume. Hemodynamics presented were averaged over 3 respiratory cycles in accordance with current guidelines at rest and exercise (Kovacs et al., 2017). The systemic oxygen extraction ratio was calculated as (CaO_2_−CvO_2_)/CaO_2_.

### 2.3 O_2_ pathway analysis

The sequence of oxygen transport and utilization beginning at the nares to mitochondrial consumption was characterized by analyzing the invasive hemodynamic data through a set of equations that govern oxygen transport and utilization (Houstis et al., 2018; Howden et al., 2021). The specific parameters that were quantified include alveolar ventilation (V̇_A_), lung diffusion capacity for O_2_ (D_L_), cardiac output (Q̇), hemoglobin concentration (Hb), skeletal muscle diffusion capacity for O_2_ (D_M_), and mitochondrial oxidative phosphorylation capacity (Vmax). This analysis was performed by the methods of Howden et al. (2021) and is available on https://bakersportscardiology.shinyapps.io/fitoxy/.

As detailed later in the manuscript to calculate V̇O_2_ deficit recovery (VDR), we normalized individual defects in the pathway. We did this by applying the peak predicted V̇O_2_, obtained by the Wasserman formula (Wasserman et al., 1987), to linear regression models formulated by Houstis et al. (2018) and Dhakal et al. (2015) from the control group. We verified the validity of these models by comparing to our controls with linear regression. We used the equations derived from Houstis et al. (2018) for VDR analysis since this data is already published and represents a larger population (*N* = 55).

### 2.4 Histopathologic analysis of skin biopsy results

Six patients on the PASC arm were referred to our neurology colleagues to undergo skin biopsies based on similarities to CFS/ME (Joseph et al., 2021) primarily based on patient request. The samples were stained with polyclonal rabbit anti‐protein‐gene‐product 9.5 antibody and were analyzed under bright‐field immunohistochemistry to determine the nerve fiber density of epidermal nerves. Skin biopsies were obtained at two sites on the outer lower leg and thigh as previously described (Raicher et al., 2022).

### 2.5 Statistical analysis

Continuous data are expressed as median [25, 75 percentile]. Categorical data are expressed as counts and percentages. The Kruskal–Wallis test was used to test for intergroup differences. Post hoc analysis was done using the Bonferroni test to correct multiple comparisons. Statistical analyses were performed using SPSS software (version 28.0, IBM, Armonk, NY). Statistical tests were 2‐sided, and a *p* value <0.05 was considered statistically significant. The data may be shared upon request. Requests will be reviewed by a data access committee in accordance with University of Arizona policies, and approved applicants must sign a data use agreement.

## 3 RESULTS

### 3.1 Characteristics of the study population

The subjects from the three different subgroups; PASC (*N* = 15), CFS/ME (*N* = 11), and Controls (*N* = 11) were similar in age, but there was a statistically higher BMI among the CFS/ME and PASC groups compared to controls (Table 1). There were also more women in all three arms of the study population compared to men. Table 1 demonstrates that resting hemodynamics were comparable in all three groups. Pulmonary function testing was within normal age and sex‐reported reference values. CT scans were notable for bronchial thickening in one CFS/ME subject and scattered ground glass in two PASC subjects. Transthoracic echocardiography reported no notable abnormalities. There were no shunts detected on echocardiography in any subjects. CMR on the PASC subjects also showed no notable abnormalities. SPECT ventilation/perfusion demonstrated small peripheral subsegmental perfusion defects in 3 PASC subjects.

**TABLE 1:** Demographics and resting hemodynamics.

Characteristic	Control	PASC	CFS	*p* Value
Age, years	41 [33, 72]	44 [21, 56]	37 [29, 45]	0.692
Female Sex	6 (66)	14 (91)	10 (88)	0.027
BMI, kg/m^2^	25 [21, 25]	31 [28, 36]	27 [24, 33]	<0.001^a^, ^b^
Time since acute COVID‐19 (months, max/min)		13 [3–50]		NA
FVC (%predicted)		95 [82, 107]	92 [77, 108]	0.90
DLCO (%predicted)		84 [78, 94]	96 [75, 103]	0.59
Resting hemodynamics				
RAP, mmHg	3 [1, 6.5]	5 [1, 6]	5 [2, 8]	0.804
mPAP, mmHg	15 [13, 17]	15 [13, 18]	15 [13, 18]	0.554
PCWP, mmHg	8 [6, 11]	8 [6, 13]	10 [7, 10]	0.431
CO, L/min	6 [4.5, 7]	5.9 [5, 6.4]	5.7 [4.1, 6.9]	0.850

*Note*: Values are median ± [25th percentile, 75th percentile] for continuous variables and *n* (%) for categorical variables. Last column *p* value represents the omnibus result from the Kruskal–Wallis test. Bonferroni used for post hoc comparisons.

Abbreviations: BMI, body mass index; CFS, chronic fatigue syndrome; CO, cardiac output; DLCO, pulmonary diffusion capacity of carbon monoxide; FVC, forced vital capacity; mPAP, mean pulmonary artery pressure; PASC, post‐acute sequelae covid; PCWP, pulmonary capillary wedge pressure; RAP, right atrial pressure.

^a^ Indicates Control versus CFS.

^b^ Indicates Control versus PASC.

PASC patients presented median 13 [range, 3–50] months since their confirmed episode of acute COVID. No subjects were hospitalized or placed on supplemental oxygen. All enrolled PASC subjects had received at least one dose of the polyvalent vaccine at the time of enrollment. Thirteen subjects were assessed before December 2021 when the predominant variant switched from delta to omicron (Thaweethai et al., 2023).

### 3.2 Standard peak exercise parameters highlight reduced cardiac index and low cardiac filling pressures

**FIGURE 1:**
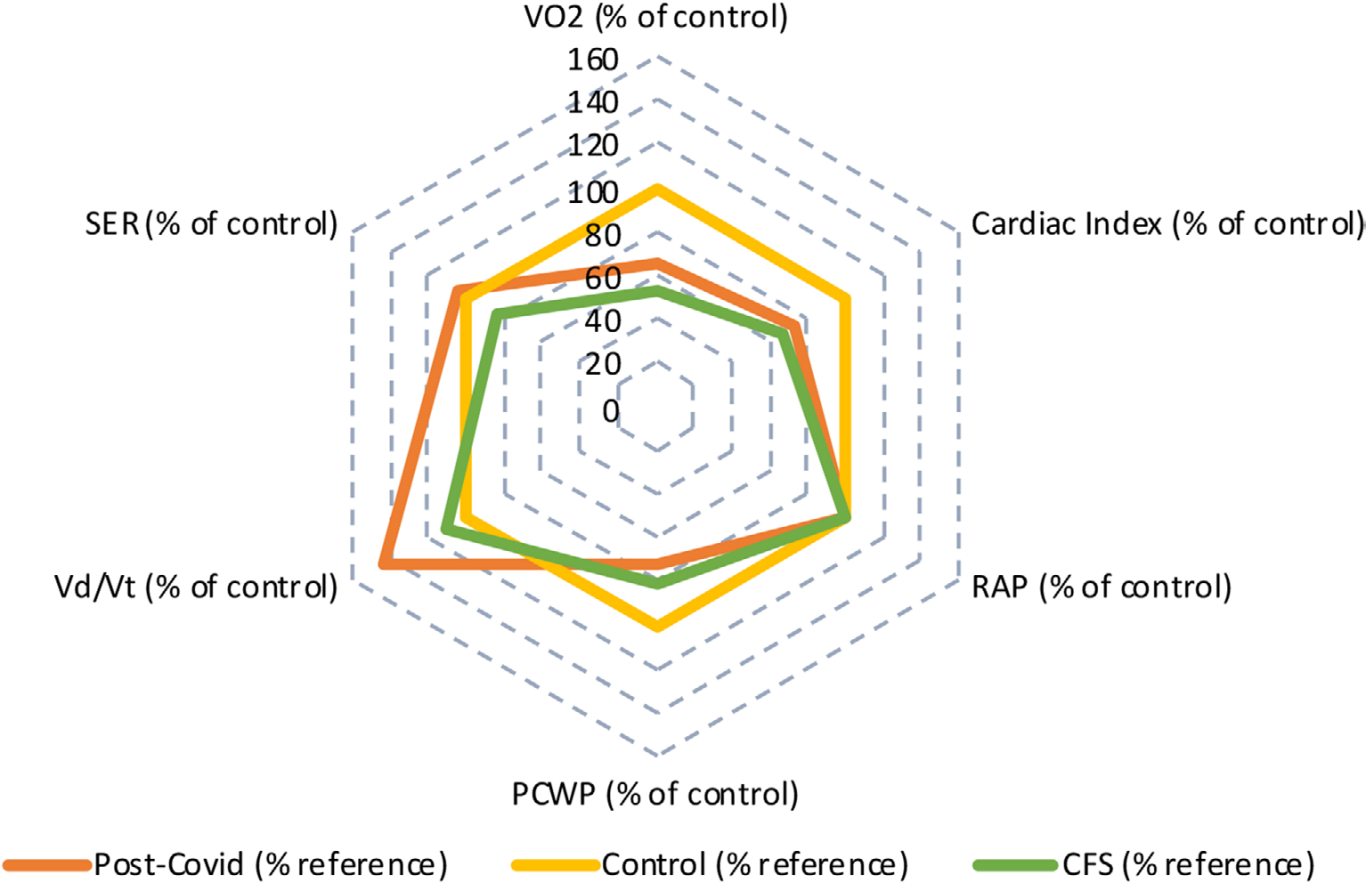
Radar plot demonstrating the following peak hemodynamics parameters among the three groups. Our control group is set at 100% as a reference line to place the other groups in context. All parameters are represented as a percentage of the control at peak exercise. PCWP, pulmonary capillary wedge pressure; RAP, right atrial pressure; SER, systemic oxygen extraction ratio; Vd/Vt, dead space to tidal volume; V̇O_2_, peak oxygen consumption.

As demonstrated by Table 2, peak V̇O_2_ and cardiac index were lower in the PASC and CFS/ME groups versus controls, with the latter being statistically significant on post hoc testing. Mixed venous (SvO_2_) was significantly higher in the CFS/ME group compared to both the control and PASC subgroups. The systemic extraction ratio was decreased for the CFS/ME group relative to controls. Although not statistically significant, cardiac filling pressures (RAP and PCWP) were both lower in PASC and CFS/ME relative to controls. As demonstrated in the radar plot (Figure 1) impairment of cardiac index appears to be a major defining parameter distinguishing PASC and CFS/ME when referenced to our control subjects.

**TABLE 2:** Peak exercise parameters by group.

Characteristic	Control	PASC	CFS	*p* Value
Peak hemodynamics				
Heart rate, bpm	127 [100, 171]	133 [125, 152]	123 [111, 137]	0.432
RAP, mmHg	4 [2.5, 7.5]	3 [1, 6]	3 [1, 6]	0.739
mPAP, mmHg	23 [21, 24]	19 [16.5, 28]	19 [18, 27]	0.367
PCWP, mmHg	13 [7.5, 13.5]	8 [6, 11]	9 [6, 15]	0.192
CO, L/min	19.2 [10.8, 22]	14.2 [12, 16.6]	12.07 [10.7, 15.2]	0.101
CI, L/min/m^2^	9.7 [7.5, 11]	7.6 [6.2, 9.5]	6.9 [5.6, 9]	0.011^a^
Blood gas analysis				
PaO_2_, mmHg	100 [86.5, 103.5]	98 [95, 107]	105 [97, 111]	0.262
PaCO_2_, mmHg	46 [37.5, 47.5]	45 [37, 57]	38 [35, 44]	0.095
PvO_2_, mmHg	26 [22, 37]	26 [24, 32]	29 [27, 31]	0.026^a^
SaO_2_%	97 [95.5, 98.5]	97 [92, 100]	99 [97, 100]	0.231
SvO_2_%	37 [32, 43]	40 [34, 49]	53 [46, 58]	0.004^a^, ^c^
HCO3^−^, mEq	23 [22, 25]	24 [23, 25]	24 [22, 26]	0.719
Lactate, mmol/L	5.3 [4.7, 9.3]	5.2 [4.7, 6.9]	2.6 [1.8, 3.2]	0.004^a^, ^c^
Cardiopulmonary exercise parameters				
V̇O_2_peak, mL/min	2048 [1608, 2376]	1361 [1015, 1677]	1115 [932, 1201]	0.003^a^, ^c^
V̇O_2_peak, % predicted	116.8 [106, 196]	68.8 [56.9, 87.7]	51.1 [48.8, 61.2]	<0.001^a^, ^b^, ^c^
V̇CO_2_peak, mL/min	2132 [1774, 2929]	1279 [921, 1695]	1170 [1064, 1227]	<0.001^a^, ^b^
SER	0.6 [0.45, 0.76]	0.59 [0.5,0.6]	0.47 [0.32, 0.54]	0.034
RQ	1.1 [1.09, 1.18]	1.1 [1.09, 1.28]	1.09 [0.98, 1.12]	0.101
V̇e/VCO_2_	31 [26.3, 37.5]	34 [31, 38]	32 [29, 35]	0.367
PetCO_2_	30.72 [25.5, 35.7]	32 [29, 37]	34 [33, 39]	0.472
Vt	1273 [994, 1400]	1369 [1246, 1718]	1273 [994, 1400]	0.128
P_ET_CO_2_, mmHg	29.76 [24, 33]	22.5 [23, 28]	27 [25, 30]	0.309
Vd/Vt	28.3 [8.9, 44.8]	40.6 [24.8, 46.5]	31.5 [23.5, 35.3]	0.194
Oxygen pathway analysis				
Q̇, L/min	19.3 [13.4, 55.8]	15 [12.3, 19]	12.6 [10.1, 17.4]	0.160
D_m_O_2_ mL/min/mmHg	49.8 [40.6, 55.4]	35.5 [26.1, 43.9]	25.9 [21.6, 27.8]	<0.001^a^, ^b^, ^c^
D_L_O_2_ mL/min/mmHg	41.5 [22.8, 53.3]	21.5 [16.4, 25]	15.8 [12.7, 19.5]	0.034
Hemoglobin, g/dL	14.9 [14.5, 15]	14.5 [13.8, 15.5]	14.1 [13.7, 14.8]	0.822
V̇a, L/min	51 [41, 58]	33 [31, 43]	30 [29, 44]	0.004
V̇maxO_2_ mL/min	3686 [2906, 4276]	2450 [1827, 3019]	2007 [1677, 2161]	0.003^a^, ^b^, ^c^

*Note*: Values are median ± [25th percentile, 75th percentile] for continuous variables and *n* (%) for categorical variables. Last column *p* value represents the omnibus result from the Kruskal–Wallis test. Bonferroni used for post‐hoc comparisons.

Abbreviations: C.I., cardiac index; CFS, chronic fatigue syndrome; CO, cardiac output; D_L_, lung diffusion capacity for O_2_; D_M_, skeletal muscle diffusion capacity for O_2_; HCO_3_, bicarbonate; mPAP, mean pulmonary artery pressure; PaCO_2_, partial arterial pressure of carbon dioxide; PaO_2_, partial arterial pressure of oxygen; PASC, post‐acute sequelae covid; PCWP, pulmonary capillary wedge pressure; P_ET_CO_2_, partial pressure of carbon dioxide in the expired air in mmHg; PvO_2_, partial venous pressure of oxygen; Q̇, calculated cardiac output; RAP, right atrial pressure; RQ, respiratory quotient; SaO_2_, arterial oxygen saturation; SvO_2_, venous oxygen saturation; V̇a, alveolar ventilation; V̇CO_2_, maximal carbon dioxide production; V̇e/VCO_2_, ventilation/carbon dioxide production; V_max_, mitochondrial oxidative phosphorylation capacity; VO_2_, maximal oxygen consumption; Vt, tidal volume; Vt/Vd, dead space ventilation.

^a^ Indicates Control versus CFS.

^b^ Indicates Control versus PASC.

^c^ Indicates CFS versus PASC.

### 3.3 Exercise O_2_ pathway parameters demonstrate defects in multiple components

Excluding hemoglobin, all oxygen parameter pathways were lower in the CFS/ME and PASC groups compared to the control group (Table 2). D_M_ and V_max_ also distinguished PASC versus CFS/ME, with CFS/ME showing more impairment in these parameters.

**FIGURE 2:**
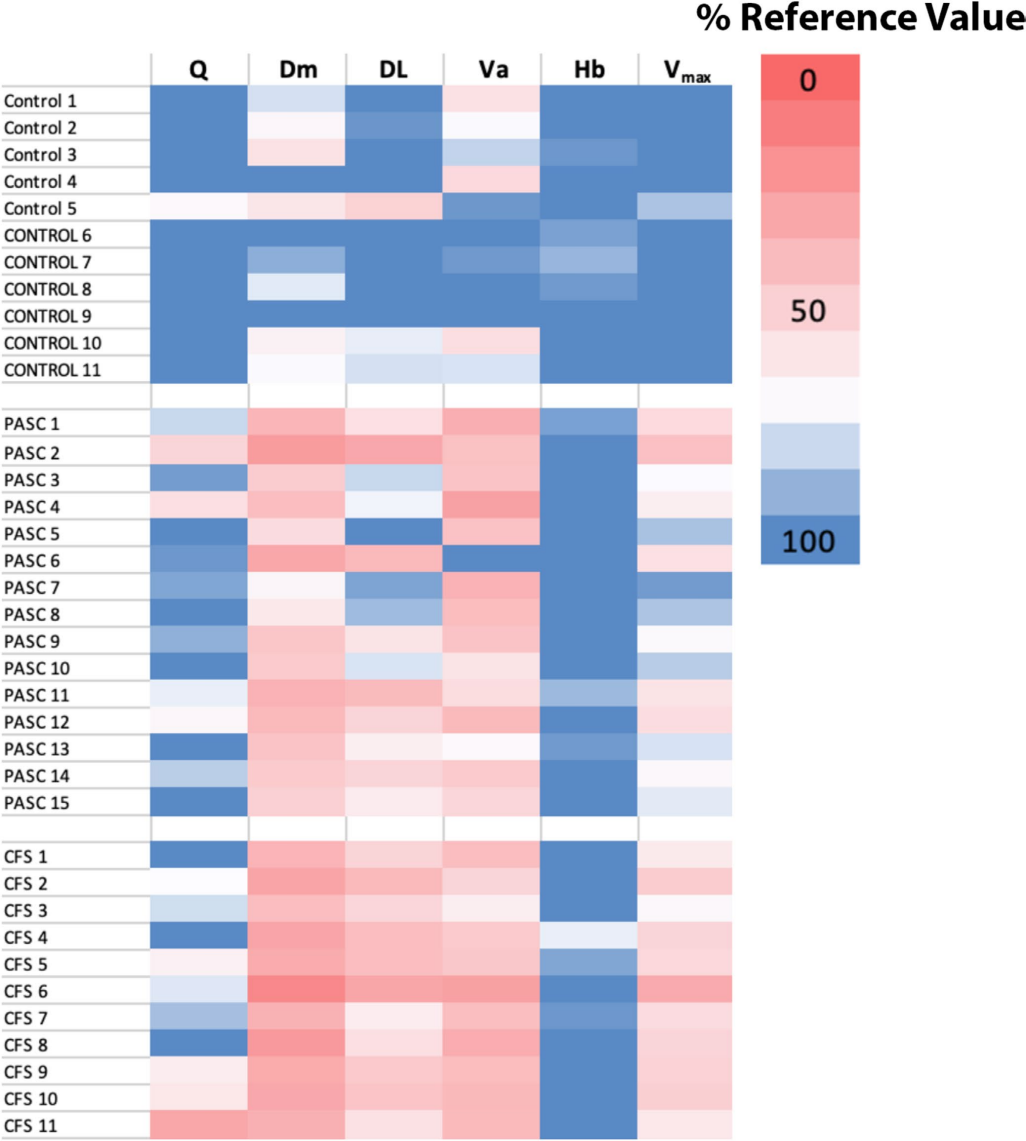
Heat map showing the percentage predicted of the six oxygen pathway parameters for Control, PASC, and CFS groups, respectively. Predicted values are generated from the control group from Houstis et al. (2018). The legend represents the corresponding color indicating % of the reference population. Each row represents an individual patient. Controls in ALL CAPS represent health controls from the Belgian cohort, while others are dyspneic controls from the University of Arizona.

Figure 2 demonstrates a heat map of individual subjects denoting the percentage predicted using the control group of Houstis et al. (2018) for each O_2_ pathway parameter. There are similarities in the underlying multicomponent pathophysiology amid PASC and CFS/ME. Notably, D_M_, D_L_, and V̇a are reduced in the PASC and CFS/ME subgroups to similar degrees.

### 3.4 Peak V̇O_2_ deficit recovery: Skeletal muscle O_2_ diffusion appears to contribute most to reduced V̇O_2_

**FIGURE 3:**
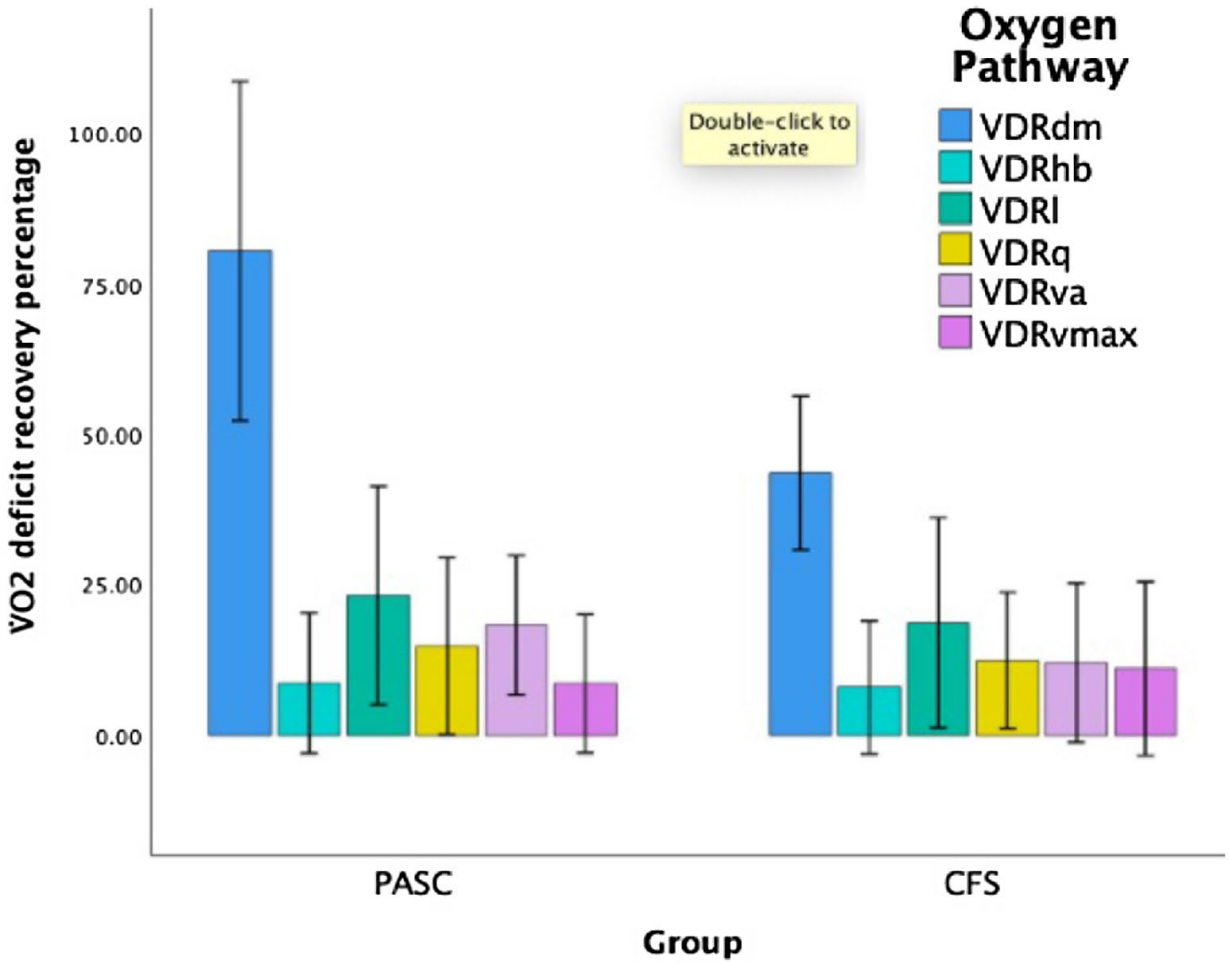
V̇O_2_ deficit recovery percentage for oxygen pathway parameters. This demonstrates the boost in V̇O_2_ after normalizing for a specific abnormal pathway. For example, cardiac output (Q̇) was normalized, and the expected improvement in peak V̇O_2_ was calculated while holding other parameters unchanged. This value is then expressed as a percentage of V̇O_2_ deficit. This is denoted as V̇O_2_ deficit recovery coefficient for cardiac output (VDRq). VDRdm, V̇O_2_ deficit recovery coefficient for skeletal muscle oxygen diffusion; VDRhb, V̇O_2_ deficit recovery coefficient for hemoglobin; VDRl, V̇O_2_ deficit recovery coefficient for pulmonary O_2_ diffusion; VDRq, V̇O_2_ deficit recovery coefficient for cardiac output; VDRva, V̇O_2_ deficit recovery coefficient for alveolar ventilation; VDRvmax, V̇O_2_ deficit recovery coefficient for mitochondrial oxidative phosphorylation. Data are presented as median (95% confidence interval).

We assessed the extent of V̇O_2_ improvement by sequentially normalizing a single defect in the oxygen extraction pathway, V̇O_2_ deficit recovery (VDR), to determine the impact of a potential targeted therapeutic approach to each O_2_ pathway parameter (Houstis et al., 2018). For instance, cardiac output was normalized for a subject while all other parameters were left unchanged. Peak V̇O_2_ was subsequently calculated for this individual denoting the boost in V̇O_2peak_ after sequentially normalizing each element for each specific abnormal pathway. These values were expressed as a percentage contribution to normalizing peak V̇O_2_. This is denoted as V̇O_2_ deficit recovery coefficient (VDRq). As Figure 3 demonstrates, we noted that normalizing D_M_ had the greatest improvement in V̇O_2_ deficit recovery followed by D_L_. Both CFS/ME and PASC demonstrate a similar pattern of V̇O_2_ deficit recoveries. Normalization of other parameters contributed <10% to reduced V̇O_2peak_.

### 3.5 Small fiber neuropathy represents a potential link to exercise intolerance among some PASC subjects

**FIGURE 4:**
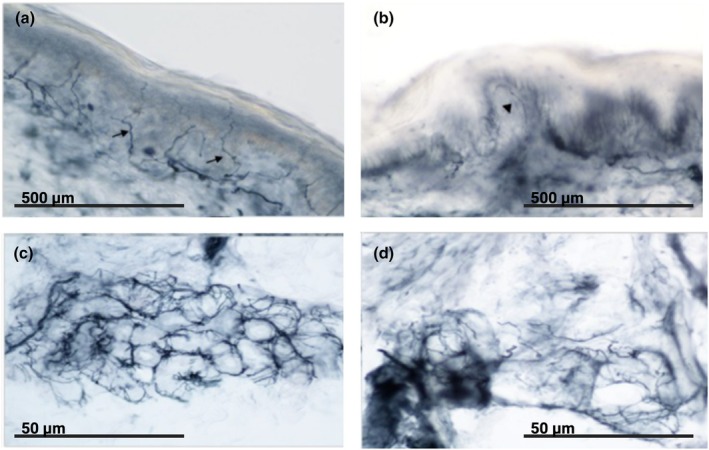
Skin biopsy immunostaining with protein‐gene‐product 9.5 antibody staining. Skin biopsy with bright‐field immunohistochemistry in 50 μm sections stained with polyclonal rabbit anti‐protein‐gene‐product 9.5 antibody. Image (a) represents a normal subject, an even distribution of epidermal nerves with slight varicosities (arrows). Image (b) is from the left upper thigh of the patient, showing decreased enervation in the epidermis and reduced axonal density (arrow). Image (c) depicts the neural innervation of a sweat gland in a subject without small fiber neuropathy. Image (d) from our patient shows reduced density of the nerves of her sweat gland.

Nerve conduction and electromyography were normal in all patients that underwent skin biopsy. Five of the six patients demonstrated findings consistent with a small fiber neuropathy. Figure 4 (a) compares the normal nerve distribution in a control subject to (b) which highlights the decreased innervation and reduced axonal density in one of our patients. Similarly, (c) shows neural innervation of a sweat gland in a control patient without small fiber neuropathy, and this is contrasted by (d) which shows small fiber neuropathy and reduced sweat gland nerve fiber density in the patient. The O_2_ pathway parameters were similar in these patients to those who did not undergo biopsy.

## 4 DISCUSSION

This study is the first to highlight parallels in the underlying pathophysiological mechanisms of PASC and CFS/ME using oxygen pathway analysis. When O_2_ pathway analysis is used, multisystem defects are evident, highlighting the phenotypic heterogeneity of PASC and CFS/ME. Of the defects, the pattern of peripheral oxygen diffusion (D_M_) appears to relate most prominently to V̇O_2_. Our results support the theory that a primary defect is consistent with an abnormal peripheral neuromuscular response to exercise. Small fiber neuropathy represents one potential link between this neuromuscular response and patient symptoms.

Although our PASC cohort reflects a bias toward exertional dyspnea and fatigue as primary symptomatology burdens, this cohort represents the most common presenting phenotype (Thaweethai et al., 2023). These subjects are characterized by normal or minimally affected standard resting tests such as echocardiography, CT chest, and pulmonary function testing. MRI does demonstrate persistent inflammatory changes of multiple organ systems, but these changes do not relate well to exercise incapacity or symptoms (Cassar et al., 2021). Our data confirm that standard resting hemodynamics are also similar between controls and PASC. This finding indicates that cardiopulmonary exercise testing may be an important diagnostic tool for phenotyping PASC.

When examining standard iCPET measurements outside of the O_2_ pathway, CFS/ME and PASC demonstrate reduced cardiac index relative to controls. This is in the absence of elevated cardiac filling pressures or pulmonary artery pressure indicating heart failure is an unlikely cause. In keeping with the lack of a rise in right atrial pressure with exercise, this may be considered “preload insufficiency” (Joseph et al., 2023). Preload insufficiency is typically detected on upright iCPET although the diagnostic criteria are not universally accepted (Tooba et al., 2021). However, preload insufficiency provides a possible relationship between exercise hemodynamics and the orthostatic symptoms through either dysautonomia (Rao et al., 2022) or cardiac deconditioning (Edward et al., 2023). Postural orthostatic tachycardia syndrome (POTS) is a frequent complication of both CFS and PASC and may represent a pathophysiological link to preload insufficiency (Rao et al., 2022).

In contrast to standard iCPET parameters, the O_2_ pathway highlights multiple organ systems involved in the exercise pathophysiology of both PASC and CFS/ME. Both the lung (D_L_ and V̇a) and peripheral neuromuscular (D_M_ and V_max_) systems appear to be affected when compared to the controls. Microclots resistant to fibrinolysis have been found in the plasma of patients with PASC (Pretorius et al., 2021). It is possible that retained (from acute COVID) or newly formed microclots cause local ischemia and inflammation. This phenomenon could account for an increase in dead space and a drop in V̇a like that seen in patients with chronic thromboembolism (Howden et al., 2021). D_L_ was less affected in our patients, likely accounting for the lack of desaturation with exercise relative to the patients in that study (Howden et al., 2021). Lung SPECT/CT, an imaging modality sensitive to microvascular disease, has demonstrated abnormalities in PASC (Piskac Zivkovic et al., 2023) was also abnormal in most of our patients tested. It is possible that microclots diffusely affect the body (Ajčević et al., 2023; Cassar et al., 2021) in PASC and CFS/ME (Nunes et al., 2022) but only those organs active during exercise reveal themselves affecting V̇O_2_. Theories as to the link between microclots and small fiber neuropathy as well as POTS have been published (Kell & Pretorius, 2023).

The V̇O_2_ deficit recovery (VDR) relates individual defects of the O_2_ pathway to improvements in V̇O_2_. This allows us to examine how potential treatment targets may influence patient function. VDR analysis showed that the primary component of exercise limitation to be D_M_, diffusion of oxygen at the skeletal muscles. D_M_ is a composite parameter that reflects the limitations to O_2_ transport from hemoglobin to mitochondria, including distributional blood flow (Nyberg & Jones, 2022). A putative hypothesis is that SFN induced dysautonomia has modified regional blood flow and resulted in a defect in coupling of flow to working muscle (Singh et al., 2021). This phenomenon has been characterized in other disease states (Zamani et al., 2020). Alternatively, amyloid deposits, inflammatory change, and reduced capillary density may play a role, as has been seen in muscle biopsies (Appelman et al., 2024; Aschman et al., 2023). Either of these proposed hypotheses can alter the skeletal muscle milieu resulting in an enhanced ergoreflex to the brain (Rischard et al., 2024; Sze et al., 2022) resulting in hyperventilation (Motiejunaite et al., 2021) and dysfunctional breathing (Mancini et al., 2021) amplifying the symptoms of dyspnea.

The similarities between PASC and CFS/ME have previously been reported (Sukocheva et al., 2022). These studies highlight the similarities in oxygen extraction/utilization as indicated by a reduced arterio‐venous O_2_ content difference and impaired systemic O_2_ extraction in both conditions (Joseph et al., 2021; Singh et al., 2021; Vermeulen & Vermeulen van Eck, 2014). For the first time, we have highlighted the similarities using the O_2_ pathway analysis. The pattern of organ system involvement is quite similar between the PASC and CFS/ME groups in our heat maps and VDR highlighting D_M_ as a therapeutic target. The groups differ only in the magnitude of involvement, with a trend toward worse disease in the CFS/ME group. Microclots are proposed to play a role in both conditions, providing a shared pathophysiologic link to impaired D_M_ (Nunes et al., 2022). Previous research has also highlighted the role of mitochondrial dysfunction in both PASC (Appelman et al., 2024) and CFS/ME (Rutherford et al., 2016). V_max_, an estimate of oxidative phosphorylation, carries some assumptions (Houstis et al., 2018) and may underestimate the role of mitochondrial dysfunction in functional limitation. This data collectively reinforces the idea that the peripheral neuromuscular system is a major player in both conditions, but that significant knowledge gaps remain.

We were not able to replicate an abnormally reduced systemic oxygen extraction (SER) ratio in both PASC and CFS/ME, as has been seen in previous studies (Mancini et al., 2021; Singh et al., 2021). This could be related to a combination of both reduced cardiac output and SER in our population. When there are combined defects limiting VO_2_, the Fick components can be difficult to interpret. For example, the normal physiological response to reduced cardiac output is an increase in Ca‐vO_2_ (and SER) due to enhanced extraction and increased hemoglobin muscle capillary transit time. Thus, a “normal extraction” in this context can be abnormal. Because these variables are interdependent, they are difficult to interpret in isolation. The O_2_ pathway resolves this by using physiologically and mathematically non‐coupled variables, and the VDR enables comparison to expected reference values. This allows identification of the dominant contributor to impaired VO_2_ even when multiple systems are involved. Consequently, Dm can be abnormal even if SER is preserved, as it is more sensitive to subtle impairments and can be interpreted independently.

One prevailing theory linking PASC to CFS/ME is deconditioning (Edward et al., 2023; Naeije & Caravita, 2021); however, recent reviews have refuted this explanation (Durstenfeld et al., 2022; Singh et al., 2021). Our data further support prior work suggesting alternative mechanisms beyond deconditioning. While deconditioning can reduce cardiac output, the dysautonomia and preload insufficiency observed in PASC and CFS/ME appear mechanistically distinct (Dani et al., 2021; Joseph et al., 2022). Muscle biopsy findings also point to abnormalities unlikely to result from deconditioning (Appelman et al., 2024; Aschman et al., 2023). PASC has also occurred in highly trained individuals (Rao et al., 2022), including three collegiate athletes in our cohort. Additionally, exertional intolerance in both conditions is often accompanied by symptom clusters such as “brain fog,” (Thaweethai et al., 2023) which are not characteristic of deconditioning. Together, these findings suggest that while deconditioning may contribute in some cases, it is unlikely to be the primary driver, and the mechanisms underlying PASC and CFS/ME are likely multifactorial.

One of the limitations in our data is the small, single‐center cohort. We present our data as exploratory in nature, potentially adding to a confluence of evidence from other cohorts. This may provide direction for focused analysis from other studies where resources are limited, or testing is not as sophisticated as iCPET. Other limitations deserve mention. We describe defects in the D_M_ pathway globally, but some studies have demonstrated D_M_ to be normal when evaluated at the level of contracting skeletal muscle (Zamani et al., 2020). Further, we did not describe how multiple comorbid O_2_ pathway defects may affect V̇O_2_ which limits our ability to draw conclusions about therapeutic targets. Also, our SFN sample was self‐referred and could represent a sub‐phenotype rather than a unifying theory of reduced V̇O_2_. Lastly, we have controls from two different populations that are not matched to the disease cohort. However, given that the study requires invasive testing with risk to the subjects, a group of controls diverse enough for matching is difficult.

In conclusion, the functional limitations of PASC and CFS/ME are not evident at rest and therefore require exercise testing to disclose. PASC and CFS/ME share multicomponent, multi‐organ pathophysiology with the predominant component being the contracting neuromuscular system. Knowledge gaps this study has highlighted are understanding the underlying mechanisms of preload insufficiency, perfusion‐metabolism uncoupling at the skeletal muscle, and hyperventilation/dysfunctional breathing. Future research can be narrowed to these components as potential therapeutic targets.

## AUTHOR CONTRIBUTIONS

F.P.R., M.I., and G.C.: study design; F.P.R., M.I., and S.K.: patient recruitment, care, and follow‐up; F.P.R., M.I., and S.K.: rest and exercise hemodynamic core interpretation; S.J., F.P.R., M.I., and S.K.: data collection, maintenance, and analysis; T.L.: pathological analysis of skin biopsy samples; S.J. and F.P.R.: statistical analysis; F.P.R. and S.J.: drafted the original manuscript; F.P.R., M.I., S.K., G.C., E.H., S.R., and T.L.: critical revision of the manuscript for important intellectual content; F.P.R.: principal investigator, had access to the study data and takes full responsibility for the integrity and accuracy of the data.

## FUNDING INFORMATION

No funding information provided.

## CONFLICT OF INTEREST STATEMENT

Dr. Rischard reports no direct conflicts related to this manuscript. His general disclosures include consulting relationships with Acceleron/Merck and United Therapeutics. He receives research support from the NIH, NHLBI, Ismed, United Therapeutics, Bayer, Merck, Janssen, Keros, and Aerovate. Dr. Levine has a financial interest in Corinthian Reference Labs and CND Life Sciences.

## ETHICS STATEMENT

The study was approved by the University of Arizona Institutional Review Board, approval number 1100000621, and all participants provided written informed consent in accordance with the Declaration of Helsinki.
